# Left ventricular systolic function in subjects conceived through assisted reproductive technologies

**DOI:** 10.3389/fcvm.2023.1059713

**Published:** 2023-03-02

**Authors:** Franziska Sciuk, Theresa Vilsmaier, Marie Kramer, Magdalena Langer, Brenda Kolbinger, Pengzhu Li, André Jakob, Nina Rogenhofer, Robert Dalla-Pozza, Christian Thaler, Nikolaus Alexander Haas, Felix Sebastian Oberhoffer

**Affiliations:** ^1^Division of Pediatric Cardiology and Intensive Care, University Hospital, LMU Munich, Munich, Germany; ^2^Division of Gynecological Endocrinology and Reproductive Medicine, Department of Obstetrics and Gynecology, University Hospital, LMU Munich, Munich, Germany

**Keywords:** assisted reproductive technologies, pediatrics, left ventricular systolic function, ejection fraction, M-mode echocardiography, two-dimensional speckle tracking echocardiography

## Abstract

**Background:**

Over the past decades, assisted reproductive technologies (ART) have gained remarkable influence in the treatment of infertility and account for more than 2 % of births in European countries nowadays. Accumulating evidence suggests ART to cause cardiovascular alterations, including left ventricular (LV) dysfunctions, within its offspring. The aim of this study was to assess LV systolic function in subjects conceived through ART in comparison to spontaneously conceived peers.

**Methods:**

For the assessment of LV morphology and LV function, M-Mode echocardiography, pulsed wave Doppler and two-dimensional speckle tracking echocardiography (2DSTE) were applied. LV ejection fraction (EF) and fractional shortening (FS) were assessed in M-Mode and calculated by Teichholz formula. EF was additionally assessed semiautomatically through 2DSTE.

**Results:**

In total, 64 ART subjects and 83 spontaneously conceived controls with no significant differences in age (12.52 ± 5.64 years vs. 13.20 ± 5.95 years, *p* = 0.486) and sex were included in the analysis. In the ART cohort, significantly lower values were observed for M-Mode assessed EF (63.63 ± 5.17 % vs. 65.35 ± 5.10 %, *p* = 0.046) and FS (34.26 ± 3.87 % vs. 35.60 ± 3.84 %, *p* = 0.038). However, after the adjustment for birth weight percentile and gestational age, M-Mode assessed EF and FS displayed no significant differences between both groups. LV morphology and remaining systolic function parameters, such as mitral annular plane systolic excursion, aortic velocity time integral, global peak longitudinal strain and 2DSTE measured EF, were comparable between both groups.

**Conclusion:**

This study suggests a lower LV systolic function in ART subjects, visualized by significantly lower values for M-Mode assessed EF and FS, compared to spontaneously conceived peers. The clinical relevance of these findings has to be investigated as the above-mentioned parameters were in normal reference range. In addition, LV systolic function parameters evaluated by other echocardiographic imaging modalities were comparable between both groups. Therefore, further studies will be required to evaluate the influence of ART on LV systolic function and cardiovascular morbidity in the future.

## Introduction

More than 40 years ago, the delivery of Louise Brown – the first child to be successfully conceived through assisted reproductive technologies (ART) – was reported (1). Nowadays, ART is widely established and facilitates conception in infertile couples all around the globe (2). The increasing popularity of ART has led to the birth of more than eight million individuals worldwide using techniques such as *in vitro* fertilization (IVF) or intracytoplasmic sperm injection (ICSI) (3). Nowadays, ART accounts for more than 2 % of births in European countries (4).

In the past, some studies have indicated a link between ART and an increased cardiovascular morbidity leading concerns to be raised about the long-term health outcome within ART offspring (5–8). IVF and ICSI treatments comprise controlled ovarian hyperstimulation, oocyte retrieval, fertilization, embryo culture and embryo transfer, including luteal support (9). As the early embryo displays a distinct vulnerability to environmental stimuli, ART is suggested to modify embryogenic development, potentially leading to the onset of chronic disease (10). In addition, pregnancies following ART may be associated with pregnancy induced hypertension, gestational diabetes, prematurity and low birth weight, all of which can *per se* negatively affect the peri- as well as the postnatal outcome (5, 11). Multiple studies described premature vascular ageing, arterial hypertension and cardiac remodeling in the ART offspring (12–14). Further, the alteration of LV diastolic function in ART subjects compared to spontaneously conceived controls is described in the literature (8). Alterations in LV diastolic function often precede detectable changes in LV systolic function (15, 16). To the best of our knowledge, limited data on LV systolic function is available for ART offspring. Prior pediatric studies that assessed LV morphometry and LV systolic function in ART subjects demonstrated inconsistent results (7, 17–19). Given the rising demand of ART in modern society and its immense epidemiologic health impact, further research is required evaluating the cardiac risk profile within the ART population. Hence, this study aimed to assess LV systolic function in ART subjects compared to spontaneously conceived peers by applying different echocardiographic methodologies.

## Methods

### Ethical approval

This study was conducted in accordance with the ethical standards of the Declaration of Helsinki. Approval for this study was obtained on the 27th of December 2020 by the Ethics Committee of the Medical Faculty of the Ludwig Maximilians University (LMU) Munich (project number: 20–0844). All participants gave written informed consent before the examination. For minors, written informed consent was additionally received from parents or legal guardians.

### Study population and study design

Study participants were recruited in cooperation with the Division of Gynecological Endocrinology and Reproductive Medicine, Department of Obstetrics and Gynecology, University Hospital, LMU Munich (Munich, Germany). Families with ART-children were contacted *via* letter. Parents and children that agreed to participate were invited to our pediatric cardiology outpatient clinic for a comprehensive cardiovascular examination. The control group was recruited *via* public calls within the greater Munich (Germany) area and was matched by age and sex with ART subjects. Only healthy, spontaneously conceived peers without known cardiovascular disease were eligible for study enrollment. To evaluate the influence of age on the cardiovascular parameters studied, participants between 4 and 26 years of age were included to represent the developmental stages of childhood, adolescence, and young adulthood.

### Medical history, course of pregnancy and birth, maternal educational level, physical examination

Information on the participants’ medical history and the regular intake of medication was gathered with particular emphasis on the cardiovascular system. Further, the smoking status of all participants was assessed. The following information on the course of pregnancy and birth was obtained retrospectively by screening clinical records and questioning the participants’ parents: birth weight (g), birth height (cm), gestational age (weeks), presence of multiple pregnancy, maternal age at birth (years), maternal body mass index (BMI, kg/m^2^) at conception, presence of gestational diabetes and presence of maternal blood pressure during pregnancy ≥ 140/90 mmHg. For birth weight and birth height, percentiles (P.) were calculated according to Voigt et al. (20). Moreover, maternal educational level was evaluated according to the German educational system: no school leaving qualification (0), lower secondary school leaving certificate (1), intermediate secondary school leaving certificate (2), general qualification for university entrance (3), completed apprenticeship (4), completed university degree (5). All participants underwent a general physical examination. Body height (cm) and body weight (kg) were measured, and BMI was determined. For study participants < 18 years of age, weight classification was assessed based on BMI P. described by Kromeyer-Hauschild et al. (21). Weight classification for study participants ≥ 18 years of age was defined as follows: underweight for BMI < 18.5 kg/m^2^, normal weight for BMI ≥ 18.5 kg/m^2^ but < 25 kg/m^2^, overweight for BMI ≥ 25 kg/m^2^ but < 30 kg/m^2^, obese for BMI ≥ 30 kg/m^2^. The Mosteller formula was utilized to calculate body surface area (BSA, m^2^) (22). Systolic blood pressure (SBP, mmHg) and diastolic blood pressure (DBP, mmHg) were measured in supine position by an automated blood pressure device (Connex Spot Monitor, 901,058 Vital Signs Monitor Core, Welch Allyn, Inc., NY, United States).

### Echocardiographic examination

Echocardiographic assessment was conducted by one investigator using a Philips iE33 xMatrix or a Philips Epiq 7G ultrasound device (Philips Healthcare, The Netherlands) with a 1–5 or a 3–8 MHz sector ultrasound transducer (Philips Healthcare, The Netherlands). Three consecutive loops were recorded under constant ECG-tracking and heart rate (bpm) was determined. Images were transferred to an offline workstation (IntelliSpace Cardiovascular Ultrasound Viewer, Philips Healthcare, The Netherlands) for analysis. Analysis for two-dimensional speckle tracking echocardiography (2DSTE) was conducted on a separate workstation (QLAB cardiovascular ultrasound quantification software, version 11.1, Philips Healthcare, The Netherlands). The offline assessment of LV systolic function was performed by one investigator for both groups.

#### M-mode echocardiography

M-mode echocardiography in parasternal long axis view was applied to determine LV dimensions at the mitral valve tip at end-diastole (QRS in ECG) and at end-systole (end of T-wave in ECG). The following parameters were assessed offline (IntelliSpace Cardiovascular Ultrasound Viewer, Philips Healthcare, The Netherlands): interventricular septum thickness at end-diastole (IVSd, mm), interventricular septum thickness at end-systole (IVSs, mm), LV end-diastolic diameter (LVEDD, mm), LV end-systolic diameter (LVESD, mm), LV posterior wall thickness at end-diastole (LVPWd, mm) and LV posterior wall thickness at end-systole (LVPWs, mm). *Z*-scores of the above-mentioned cardiac structures were calculated for minor study participants according to Kampmann et al. (23). Relative wall thickness (RWT) was calculated as $RWT=\frac{2\times \mathrm{LVPWd}}{\mathrm{LVEDD}}$. LV mass (LVM, g) was calculated according to the American Society of Echocardiography guidelines (24) and indexed to BSA (LVMI, g/m^2^). LV end-diastolic volume (EDV, mL) and LV end-systolic volume (ESV, mL) were determined using M-Mode assessed LVEDD and LVESD applying the Teichholz formula (25). LV stroke volume (SV, mL) was calculated as the difference of EDV and ESV. LV ejection fraction (EF, %) was defined as $EF\;\left(\%\right)=\;\frac{SV}{EDV}\times 100$. To determine fractional shortening (FS, %), the following equation was used: $FS\;\left(\%\right)=\frac{\mathrm{LVEDD}-\mathrm{LVESD}}{\mathrm{LVEDD}}\times 100$. The diameter of the aortic root (AO, mm) at end-diastole and the diameter of the left atrium (LA, mm) at end-systole were assessed in M-Mode in parasternal long axis view. The LA/AO ratio was calculated. Mitral annular plane systolic excursion (MAPSE, mm) was assessed in apical four chamber (A4C) view on the lateral mitral valve annulus using M-Mode echocardiography. Longitudinal systolic excursion was determined through offline analysis by measuring the total displacement of the mitral annulus in relation to the apex from end-diastole to end-systole.

#### Pulsed wave Doppler

The aortic velocity time integral (aVTI, cm) was assessed in apical five chamber view through pulsed wave Doppler by placing the sample volume over the aortic valve. aVTI was manually determined in an offline analysis (IntelliSpace Cardiovascular Ultrasound Viewer, Philips Healthcare, The Netherlands) by tracing the area under the spectral curve.

#### Two-dimensional speckle tracking echocardiography

**Figure 1:**
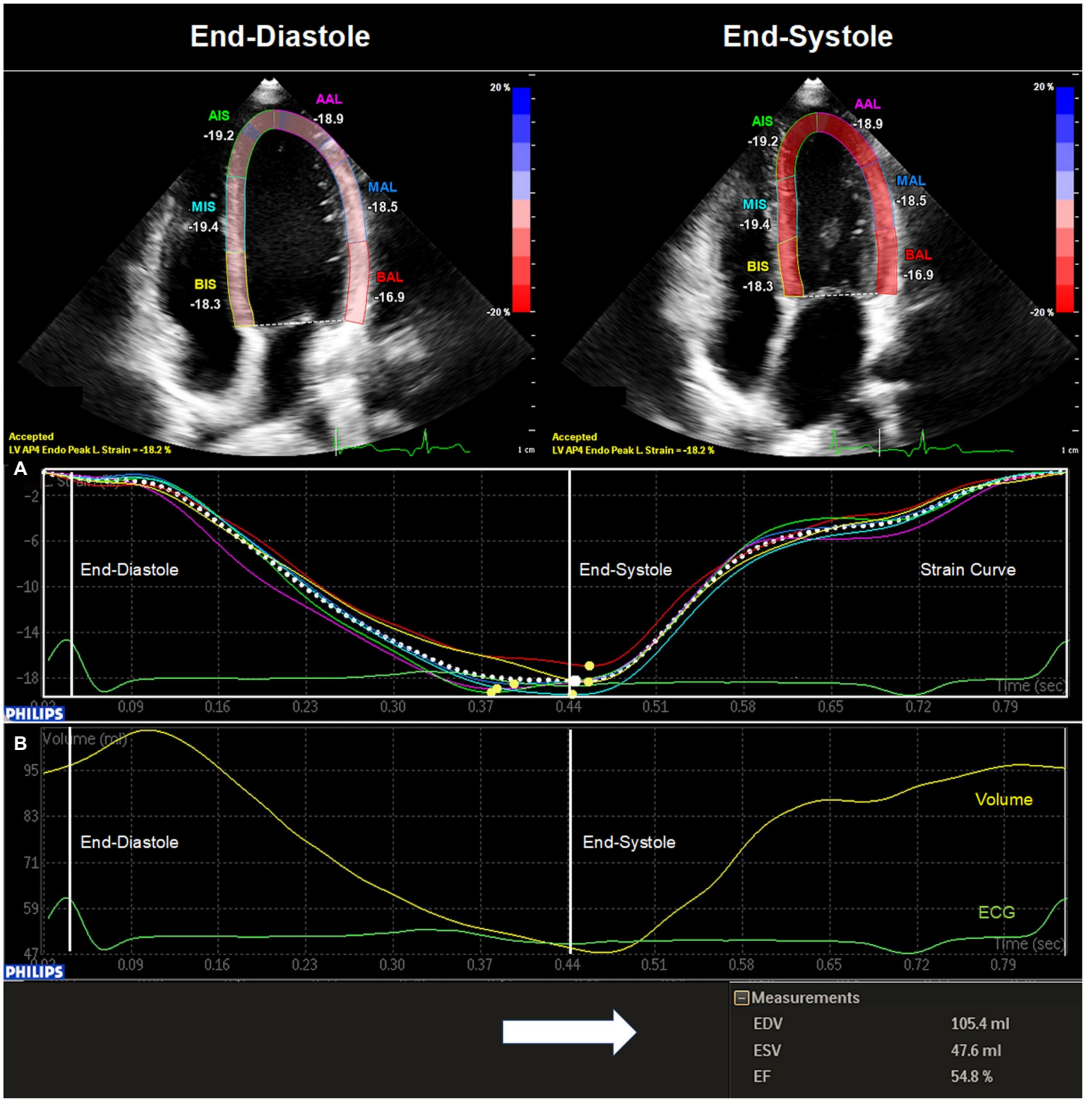
Two-dimensional speckle tracking echocardiography of the left ventricle. The image was acquired through echocardiography in apical four chamber view and transferred to an offline workstation (QLAB cardiovascular ultrasound quantification software, version 11.1, Philips Healthcare, The Netherlands) for further analysis. In end-diastole (QRS in ECG) the investigator marked the endocardium at three fixed points. The endocardium was then automatically marked at end-diastole by the software. The investigator could manually adjust the region of interest at end-diastole and at end-systole (end of T-wave in ECG) if necessary. **(A)** Global peak longitudinal strain for apical four chamber view (GPLS_A4C, %) was then automatically assessed by the software at end-systole. **(B)** In addition, a left ventricular volume curve was generated enabling the calculation of left ventricular end-diastolic volume (EDV, mL), left ventricular end-systolic volume (ESV, mL), and left ventricular ejection fraction (EF, %).

Images for 2DSTE were acquired in apical two chamber view (A2C), apical three chamber view (A3C) and A4C. In each chamber view, four loops were recorded under constant ECG tracking. Recordings were then transferred to an offline workstation (QLAB cardiovascular ultrasound quantification software, version 11.1, Philips Healthcare, The Netherlands) for further analysis. In end-diastole the investigator marked the endocardium at three fixed points. The endocardium was then automatically marked at end-diastole by the software. Myocardial speckles of the LV were then tracked by the software throughout the cardiac cycle until end-systole allowing the calculation of peak longitudinal strain for each chamber view. The investigator could manually adjust the region of interest at end-diastole and at end-systole if necessary. Global peak longitudinal strain (GPLS, %) was assessed for A2C (GPLS_A2C, %), A3C (GPLS_A3C, %), and A4C (GPLS_A4C, %) (Figure 1A). Moreover, an average of the above-mentioned values was calculated (GPLS_AVG, %). Additionally, EDV, ESV and EF were assessed semiautomatically by the software (QLAB cardiovascular ultrasound quantification software, version 11.1, Philips Healthcare, The Netherlands) in A4C (Figure 1B).

### Statistical analysis

For statistical analysis, SPSS 28 (IBM SPSS Statistics for Windows, Version 28.0. IBM Corp., Armonk, NY, United States) was used. Variables were tested for normal distribution using the Kolmogorov–Smirnov or the Shapiro–Wilk test. Normally distributed variables were analyzed using an independent *t*-test and non-normally distributed variables were compared using the Mann–Whitney-*U* test. For large sample sizes (*n* ≥ 30), data was considered normally distributed and the independent *t*-test was utilized for analysis. Data is presented as mean ± standard deviation or as median (range). Categorical variables were analyzed using the Pearson’s chi-squared test or Fisher’s exact test and are displayed in counts or percentages. For correlation analysis the Pearson correlation coefficient was chosen for normally distributed variables and the Spearman’s rank correlation coefficient for non-normally distributed variables. To adjust for the influence of birthweight P. and gestational age on LV morphology and LV systolic function, linear regression was utilized. In respect to multiple testing, the alpha level was adjusted by applying the Holm–Bonferroni correction. The level of significance was defined as *p* < 0.05.

Ten randomly selected study participants were chosen to assess intra- and interobserver variability (%) by two experienced investigators for the following echocardiographic parameters: EDV (Teichholz), ESV (Teichholz), SV (Teichholz), EF (Teichholz), FS (Teichholz), GPLS_A2C, GPLS_A3C, GPLS_A4C, GPLS_AVG, EDV (A4C), ESV (A4C), SV (A4C), and EF (A4C). The following formula: $\frac{/A-B/}{\left[\left(A+B\right)/2\right]}\times 100$ was used to calculate relative intra- and interobserver variability (%) (26).

## Results

### Patients’ characteristics

In total, 156 subjects were enrolled in the present study. Two individuals in the ART group were excluded due to history of T-cell lymphoma and cardiac surgery, respectively. One ART child was excluded based on its conception mode (gamete intrafallopian transfer). In addition, three individuals in the ART group and three individuals in the control group were excluded due to incomplete echocardiographic data assessment. In total, 147 subjects were included for the final analysis (64 individuals in the ART group, 83 individuals in the control group).

Within the ART group, 49 subjects were conceived through ICSI and 15 through IVF. One ART subject displayed with hypercholesteremia, one with hypothyroidism, one with a possible history of myocarditis and one with long QT syndrome. Further, two ART individuals were taking oral contraceptives as regular medication, one l-thyroxine and one methylphenidate. In the control group, five subjects were using oral contraceptives, one bisoprolol due to migraine and one methylphenidate. Two individuals in the control group and three in the ART group were smokers.

ART and control group did not differ significantly in age (12.52 ± 5.64 years vs. 13.20 ± 5.95 years, *p* = 0.486) and sex (37 female vs. 42 female, *p* = 0.385). No significant differences were demonstrated for anthropometric variables, SBP, DBP or heart rate between both groups. ART subjects displayed significantly lower values for birth weight, birth weight P., birth height and gestational age. Further, the ART group showed a significantly higher prevalence of multiple pregnancy and a significantly higher maternal age at birth. The remaining data on the course of pregnancy and birth as well as maternal educational level did not differ significantly between both groups. Patients’ characteristics are summarized in Table 1.

**Table 1:** Patients’ characteristics.

Variable	Control (*n* = 83)	ART (*n* = 64)	Value of *p*
Age (years)	13.20 ± 5.95	12.52 ± 5.64	0.486
Female [*n* (%)]	42 (50.60)	37 (57.81)	0.385
Body weight (kg)	44.73 ± 18.71	41.72 ± 19.44	0.343
Body height (cm)	151.94 ± 23.08	146.40 ± 21.80	0.142
BSA (m^2^)	1.36 ± 0.39	1.29 ± 0.39	0.256
BMI (kg/m^2^)	18.32 ± 3.29	18.21 ± 3.67	0.853
Underweight [*n* (%)]	5 (6.02)	4 (6.25)	1.000
Normal weight [*n* (%)]	72 (86.75)	55 (85.94)	
Overweight [*n* (%)]	6 (7.23)	5 (7.81)	
Obese [*n* (%)]	0 (0)	0 (0)	
SBP (mmHg)	110.83 ± 11.40	110.36 ± 11.78	0.807
DPB (mmHg)	67.59 ± 7.87	67.45 ± 8.69	0.920
Heart rate (bpm)	72.48 ± 13.04	72.45 ± 12.79	0.989
Smoking [n (%)]	2 (2.41)	3 (4.69)	0.653
Course of pregnancy and birth, maternal educational level			
Birth weight (g)^1^	3,414 ± 447	2,771 ± 796	<0.001***
Birth weight percentile^2^	52.55 ± 23.58	38.57 ± 24.89	0.001**
Birth height (cm)^3^	51.71 ± 2.71	49.13 ± 4.59	<0.001***
Birth height percentile^4^	55.86 ± 28.89	51.58 ± 28.51	0.400
Gestational age (weeks)^5^	38.96 ± 1.55	36.78 ± 3.90	<0.001***
Multiple pregnancy [*n* (%)]^6^	2 (2.82)	24 (43.64)	<0.001***
Maternal age at birth (years)^7^	33.08 ± 4.19	35.39 ± 3.79	<0.001***
Maternal BMI at conception (kg/m^2^)^8^	22.01 ± 2.97	23.02 ± 4.08	0.164
Gestational diabetes [*n* (%)]^9^	3 (4.29)	3 (5.56)	1.000
Maternal blood pressure during pregnancy ≥ 140/90 mmHg [*n* (%)]^10^	3 (6.67)	0 (0)	0.287
Maternal educational level^11^	4 (1–5)	5 (2–6)	0.137

ART, assisted reproductive technologies; BSA, body surface area; BMI, body mass index; SBP, systolic blood pressure; DBP, diastolic blood pressure. Maternal level of education was assessed according to the German educational system: no school leaving qualification (0), lower secondary school leaving certificate (1), intermediate secondary school leaving certificate (2), general qualification for university entrance (3), completed apprenticeship (4), completed university degree (5). Data is presented as mean ± SD if normally distributed. Categorical data is presented as *n* (%). ** Value of *p* < 0.01. *** Value of *p* < 0.001. ^1^76 controls and 63 ART subjects were included in the analysis. ^2^74 controls and 60 ART subjects were included in the analysis. ^3^76 controls and 61 ART subjects were included in the analysis. ^4^73 controls and 57 ART subjects were included in the analysis. ^5^74 controls and 60 ART subjects were included in the analysis. ^6^71 controls and 55 ART subjects were included in the analysis. ^7^81 controls and 63 ART subjects were included in the analysis. ^8^60 controls and 45 ART subjects were included in the analysis. ^9^70 controls and 54 ART subjects were included in the analysis. ^10^45 controls and 27 ART subjects were included in the analysis. ^11^49 controls and 44 ART subjects were included in the analysis.

### M-mode echocardiography and pulsed wave Doppler

**Figure 2:**
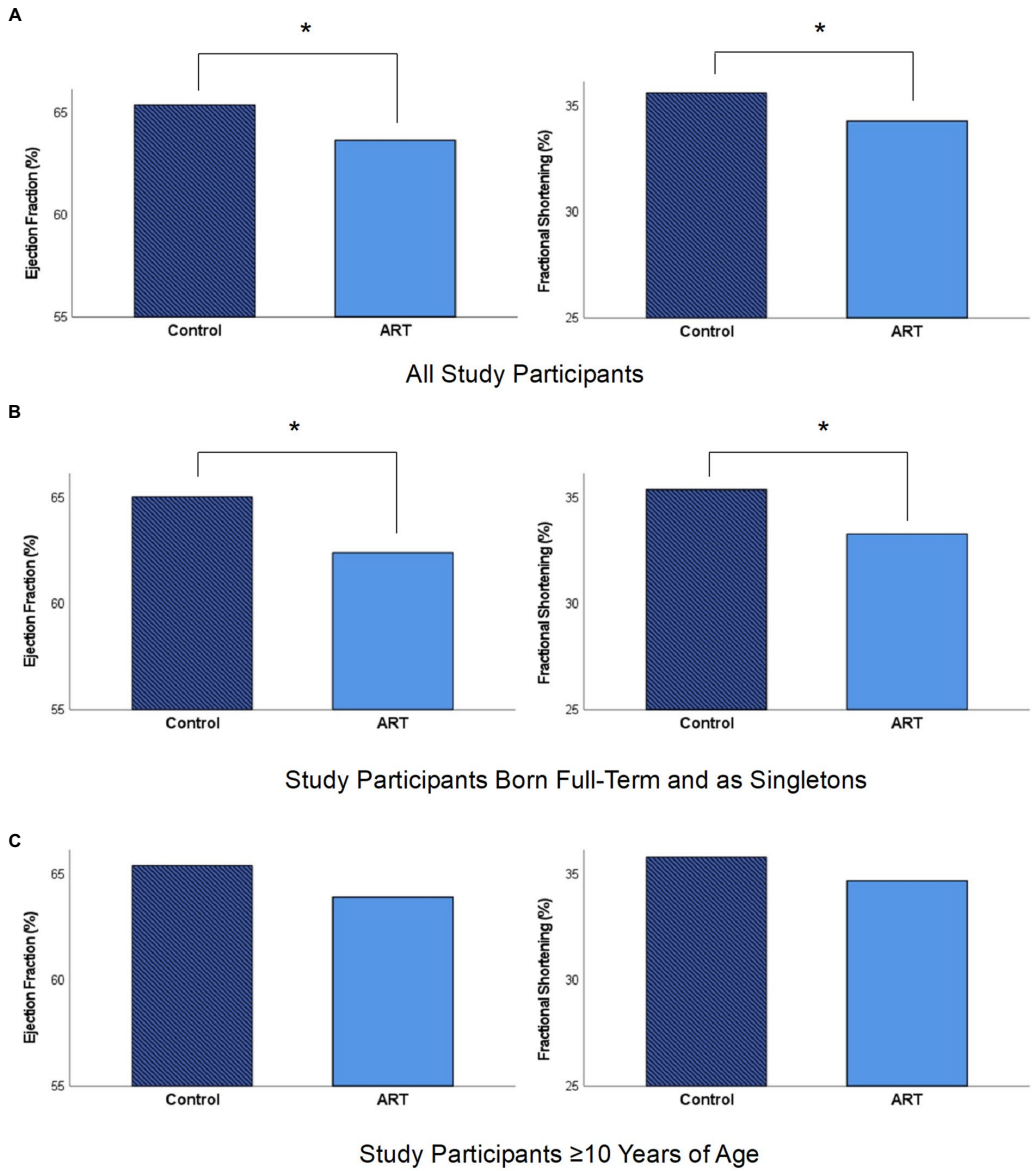
Left ventricular ejection fraction and fractional shortening assessed by *M*-mode echocardiography in the control and ART group. **(A)** Eighty-three control subjects and 64 ART subjects displayed significant differences for ejection fraction (65.35 ± 5.10 % vs. 63.63 ± 5.17 %, *p* = 0.046, adjusted *p* = 0.576) and fractional shortening (35.60 ± 3.84 % vs. 34.26 ± 3.87 %, *p* = 0.038, adjusted *p* = 0.543). **(B)** Fifty-nine control subjects and 20 ART subjects that were born on full term (≥ 37 weeks of gestation, birth height ≥ 10. P., birth weight ≥ 10. P.) and as singletons displayed significant differences for ejection fraction (65.01 ± 5.00 % vs. 62.38  ± 4.99 %, *p* = 0.046, adjusted *p* = 0.280) and fractional shortening (35.35 ± 3.77 % vs. 33.26 ± 3.67 %, *p* = 0.034, adjusted *p* = 0.224). **(C)** Fifty-two control subjects and 36 ART subjects ≥ 10 years of age displayed no significant differences for ejection fraction (65.37 ± 4.97 % vs. 63.88 ± 5.25 %, *p* = 0.182, adjusted *p* = 0.576) and fractional shortening (35.77 ±  3.74 % vs. 34.65 ± 3.97 %, *p* = 0.183, adjusted *p* = 0.570). All parameters were obtained from *M*-mode echocardiography in parasternal long axis view. * Value of *p* < 0.05.

The assessment of cardiac morphometry including LV dimensions, RWT, LVM and LA/AO revealed no significant difference between the ART and control group. Functional LV parameters such as SV, MAPSE or aVTI demonstrated no significant differences between both groups. Interestingly, EF (63.63 ± 5.17 % vs. 65.35 ± 5.10 %, *p* = 0.046, adjusted *p* = 0.576) and FS (34.26 ± 3.87 % vs. 35.60 ± 3.84 %, *p* = 0.038, adjusted *p* = 0.543) calculated by Teichholz formula showed significantly lower values within the ART group compared to spontaneously conceived controls (Figure 2A). However, after the adjustment for birth weight P. and gestational age, M-Mode assessed EF and FS displayed no significant differences between both groups. Data on LV morphometry and systolic function assessed by M-Mode and pulsed wave Doppler is provided in Tables 2, 3.

**Table 2:** Left ventricular morphometry assessed by *M*-mode echocardiography.

*Study participants < 18 years of age*				
Variable	Control (*n* = 63)	ART (*n* = 49)	Unadjusted value of *p*	Adjusted value of *p*^a,b^
IVSd *z*-score	0.18 ± 1.04	0.25 ± 1.02	0.707	>0.999
IVSs *z*-score	0.29 ± 0.80	0.11 ± 0.93	0.290	0.573
LVEDD *z*-score	−0.42 ± 1.01	−0.32 ± 1.03	0.589	>0.999
LVESD *z*-score	−0.24 ± 0.90	0.03 ± 0.91	0.123	>0.999
LVPWd *z*-score	0.25 ± 0.81	0.45 ± 0.82	0.187	0.232
LVPWs *z*-score	−0.20 ± 0.95	−0.45 ± 0.93	0.168	>0.999
*Study participants ≥ 18 years of age*				
Variable	Control (*n* = 20)	ART (*n* = 15)	Unadjusted value of *p*	Adjusted value of *p*^a,b^
IVSd (mm)	9.60 (6.70–12.00)	9.60 (7.30–10.00)	0.328	>0.999
IVSs (mm)	12.42 ± 1.76	12.07 ± 1.22	0.516	>0.999
LVEDD (mm)	46.30 ± 4.54	44.40 ± 3.11	0.174	0.738
LVESD (mm)	30.05 ± 3.27	29.20 ± 2.60	0.413	0.550
LVPWd (mm)	9.10 ± 1.44	8.91 ± 1.00	0.659	>0.999
LVPWs (mm)	12.69 ± 2.07	12.19 ± 1.72	0.450	>0.999
All study participants				
Variable	Control (*n* = 83)	ART (*n* = 64)	Unadjusted value of *p*	Adjusted value of *p*^a,b^
RWT	0.36 ± 0.07	0.37 ± 0.06	0.557	>0.999
LVM (g)	96.01 ± 44.03	88.73 ± 38.02	0.294	>0.999
LVMI (g/m^2^)	68.04 ± 16.09	67.23 ± 14.20	0.751	>0.999
AO (mm)^1^	18.16 ± 3.19	17.67 ± 2.97	0.347	>0.999
LA (mm)^1^	20.61 ± 3.85	19.89 ± 3.41	0.241	>0.999
LA/AO^1^	1.14 ± 0.15	1.13 ± 0.13	0.672	>0.999

ART, assisted reproductive technologies; IVSd, interventricular septum thickness at end-diastole; IVSs, interventricular septum thickness at end-systole; LVEDD, left ventricular end-diastolic diameter; LVESD, left ventricular end-systolic diameter; LVPWd, left ventricular posterior wall thickness at end-diastole; LVPWs, left ventricular posterior wall thickness at end-systole; RWT, relative wall thickness; LVM, left ventricular mass; LVMI, left ventricular mass index; AO, aortic root diameter; LA, left atrial diameter. Data is presented as mean ± SD if normally distributed or as median (range) if non-normally distributed. ^1^82 controls and 64 ART subjects were included in the analysis. ^a^Adjusted for birth weight percentile and gestational age. ^b^Corrected for multiple testing (Holm–Bonferroni).

**Table 3:** Left ventricular systolic function assessed by *M*-mode echocardiography and pulsed wave Doppler.

Variable	Control (*n* = 83)	ART (*n* = 64)	Unadjusted value of *p*	Adjusted value of *p*^a,b^
*M*-mode echocardiography				
EDV (mL) (Teichholz)	77.93 ± 24.82	73.38 ± 22.58	0.254	>0.999
ESV (mL) (Teichholz)	27.09 ± 9.92	26.63 ± 8.85	0.769	>0.999
SV (mL) (Teichholz)	50.84 ± 16.24	46.75 ± 15.14	0.122	>0.999
EF (%) (Teichholz)	65.35 ± 5.10	63.63 ± 5.17	0.046*	0.576
FS (%) (Teichholz)	35.60 ± 3.84	34.26 ± 3.87	0.038*	0.543
MAPSE (mm)	16.27 ± 2.75	15.67 ± 2.80	0.194	>0.999
Pulsed wave Doppler				
aVTI (cm)	23.87 ± 3.63	22.89 ± 2.81	0.076	0.196

ART, assisted reproductive technologies; EDV, end-diastolic volume; ESV, end-systolic volume; SV, stroke volume; EF, ejection fraction; FS, fractional shortening; MAPSE, mitral annular plane systolic excursion; aVTI, aortic velocity time integral. Data is presented as mean ± SD if normally distributed. * Value of *p* < 0.05. ^a^Adjusted for birth weight percentile and gestational age. ^b^Corrected for multiple testing (Holm–Bonferroni).

### Two-dimensional speckle tracking echocardiography

No significant differences in GPLS were assessed between both groups when 2DSTE was applied. Further, LV volumes and EF assessed semiautomatically through 2DSTE in A4C differed not significantly between both groups. Information on 2DSTE data is given in Table 4.

**Table 4:** Two-dimensional speckle tracking echocardiography.

Variable	Control (*n* = 83)	ART (*n* = 64)	Adjusted value of *p*	Unadjusted value of *p*^a,b^
Strain analysis				
GPLS_A2C (%)	−19.71 ± 1.43	−19.59 ± 1.52	0.621	>0.999
GPLS_A3C (%)	−19.58 ± 1.37	−19.36 ± 1.72	0.394	>0.999
GPLS_A4C (%)	−20.17 ± 1.46	−19.79 ± 1.60	0.134	>0.999
GPLS_AVG (%)	−19.82 ± 0.97	−19.58 ± 1.16	0.175	>0.999
Semiautomatic assessment of left ventricular volumes and systolic function				
EDV (mL) (A4C)	72.97 ± 27.94	64.91 ± 24.45	0.070	0.560
ESV (mL) (A4C)	34.07 ± 13.39	30.22 ± 11.96	0.072	0.348
SV (mL) (A4C)	38.90 ± 15.58	34.69 ± 13.20	0.085	0.996
EF (%) (A4C)	53.15 ± 4.66	53.44 ± 4.45	0.709	0.596

ART, assisted reproductive technologies; GPLS_A2C, global peak longitudinal strain in apical two chamber view; GPLS_A3C, global peak longitudinal strain in apical three chamber view; GPLS_A4C, global peak longitudinal strain in apical four chamber view; GPLS_AVG, averaged global peak longitudinal strain; EDV, end-diastolic volume; ESV, end-systolic volume; SV, stroke volume; EF, ejection fraction; A4C, apical four chamber view. Data is presented as mean ± SD if normally distributed. ^a^Adjusted for birth weight percentile and gestational age. ^b^Corrected for multiple testing (Holm–Bonferroni).

### Influence of perinatal conditions and age on left ventricular systolic function

Twenty ART subjects and 59 control subjects that were born on full term (≥ 37 weeks of gestation, birth height ≥ 10. P., birth weight ≥ 10. P.) and as singletons were further analyzed. No significant differences in age [9.53 (5.30–24.33) years vs. 11.95 (4.34–26.05) years, *p* = 0.388], sex (12 female vs. 28 female, *p* = 0.332) and anthropometric parameters were detected between both groups. The following M-Mode derived parameters displayed significantly lower values within ART subjects compared to controls: EF (62.38 ± 4.99 % vs. 65.01 ± 5.00 %, *p* = 0.046, adjusted *p* = 0.280), FS (33.26 ± 3.67 % vs. 35.35 ± 3.77 %, *p* = 0.034, adjusted *p* = 0.224) and SV [41.23 (22.84–77.81) mL vs. 50.86 (21.71–93.88) mL, *p* = 0.032, adjusted *p* = 0.204] (Figure 2B). The remaining parameters of LV morphology and systolic function displayed no significant differences between both groups.

Further, LV systolic function was compared in 28 ART and 31 spontaneously conceived children who were < 10 years of age [7.37 (4.41–9.82) years vs. 7.50 (4.34–9.84) years, *p* = 0.970]. Within the ART group, significantly higher values for RWT (0.36 ± 0.06 vs. 0.33 ± 0.05, *p* = 0.047, adjusted *p* = 0.256) were displayed. The comparison of 36 ART and 52 control subjects ≥ 10 years of age (16.45 ± 4.34 years vs. 16.59 ± 4.90 years, *p* = 0.891) revealed no significant differences for LV morphology and systolic function between both groups. For subjects < 10 and ≥ 10 years of age (Figure 2C), EF and FS displayed a tendency to be lower within the ART group.

### Correlation analysis

A correlation analysis was conducted for M-Mode and 2DSTE assessed EF as well as FS with age, BMI, SBP, birth weight and gestational weeks in both study groups. However, none of the above-mentioned parameters showed a significant correlation in the ART or the control group.

### Relative intra- and interobserver variability of echocardiographic variables

Table 5 summarizes data on relative intra- and interobserver variability of echocardiographic variables.

**Table 5:** Relative intra- and interobserver variability of echocardiographic variables.

Variable	Intraobserver variability (%)	Interobserver variability (%)
*M*-mode echocardiography		
EDV (Teichholz)	8.86 ± 6.09	7.97 ± 5.83
ESV (Teichholz)	5.56 ± 7.78	11.03 ± 5.97
SV (Teichholz)	15.55 ± 10.32	11.59 ± 6.50
EF (Teichholz)	6.95 ± 6.87	5.58 ± 4.77
FS (Teichholz)	9.31 ± 8.51	7.42 ± 5.64
Strain analysis		
GPLS_A2C	3.35 ± 2.28	5.90 ± 4.04
GPLS_A3C	3.22 ± 3.46	6.50 ± 3.47
GPLS_A4C	2.64 ± 1.85	5.40 ± 3.25
GPLS_AVG	1.77 ± 1.60	2.17 ± 1.89
Semiautomatic assessment of left ventricular volumes and systolic function		
EDV (A4C)	4.32 ± 3.19	6.11 ± 6.71
ESV (A4C)	4.77 ± 4.15	9.91 ± 4.16
SV (A4C)	6.28 ± 4.92	8.89 ± 9.04
EF (A4C)	4.00 ± 2.33	5.83 ± 4.14

EDV, end-diastolic volume; ESV, end-systolic volume; SV, stroke volume; EF, ejection fraction; FS, fractional shortening; GPLS_A2C, global peak longitudinal strain in apical two chamber view; GPLS_A3C, global peak longitudinal strain in apical three chamber view; GPLS_A4C, global peak longitudinal strain in apical four chamber view; GPLS_AVG, averaged global peak longitudinal strain; A4C, apical four chamber view. Data is presented as mean ± SD.

## Discussion

To our knowledge, this is one of the largest studies evaluating LV systolic function in ART subjects. Sixty-four individuals conceived through ART and 83 spontaneously conceived peers were examined using different echocardiographic imaging techniques. Within the ART cohort, a significantly lower EF and FS was demonstrated through M-Mode echocardiography suggesting a potentially lower LV systolic function. However, after the adjustment for birth weight P. and gestational age, M-Mode assessed EF and FS displayed no significant differences between both groups. In addition, the remaining parameters of LV systolic function - including MAPSE, aVTI, GPLS and EF assessed in A4C – did not differ significantly between both groups.

### Left ventricular dysfunction in the ART offspring

#### Comparison to previous studies

In the literature, multiple pediatric studies suggest unfavorable cardiovascular changes in ART children: Valenzuela-Alcaraz et al. (14) described cardiac remodeling in ART fetuses and infants visualized by more globular hearts with thicker myocardial walls as well as an impaired relaxation and functional decrease in longitudinal systolic excursion. The authors suggest these findings to be based on pressure overload. In line with this, a meta-analysis with a total of 3,034 IVF/ICSI and 872 control participants revealed significantly higher SBP (1.88 mmHg, 95 % CI 0.27–3.49 mmHg) and DBP (1.51 mmHg, 95 % CI 0.33–2.70 mmHg) levels within the ART cohort, suggesting arterial hypertension to be one of the first manifestations of vascular dysfunction in ART offspring (8). Additionally, prior studies demonstrated alterations in the vascular system of ART subjects including elevated blood pressure levels, increased pulse wave velocity, increased carotid intima-media thickness or lower flow-mediated dilation (12, 13). Overall, these findings indicate a higher risk of premature vascular ageing and increased arterial stiffness within the ART population (13). Elevated blood pressure and arterial stiffness – as observed in ART subjects – increase LV afterload pressure (27). To compensate for pressure overload, the LV undergoes structural changes including hypertrophy (14). LV hypertrophy (LVH) can translate into diastolic dysfunction visualized by impaired relaxation and filling dynamics (16). The gain of LVM due to LVH is also negatively correlated with systolic performance and a chronic excess of overload might progress into systolic dysfunction (28, 29). According to this, Cui et al. studying 764 children (6–10 years of age) reported both, lower LV systolic and diastolic function, on the ground of cardiac remodeling processes including a higher prevalence of LVH and higher values for LVMI and RWT (7). However, not all studies have come to the same conclusion. Halliday et al. conducted a study including 193 ART and 86 spontaneously conceived adults (22–35 years of age) and found no evidence for an increased vascular (e.g., SBP, DBP) or cardiometabolic risk within ART subjects (30). Bi et al. (31) reported significant changes in LV geometry in ART fetuses visualized by a more globular and enlarged LV shape as well as a reduced LV systolic deformation. Interestingly, changes in LV systolic function and geometry were no longer evident in postnatal follow-up examinations (31). Similarly, our study did not display a significant increase in SBP or DBP and no signs of load dependent cardiac remodeling were observed. LVM, LVMI, RWT were comparable between both groups. Within a sub analysis of participants <10 years of age, a significantly higher RWT was displayed within the ART cohort. Even though, no major signs of cardiac remodeling were observed in this study, a significantly lower M-Mode assessed EF was displayed in the ART cohort. Thus, LV systolic function might not only be affected by vascular dysfunction, emphasizing the potential involvement of multiple pre- or postnatal factors inducing alterations in cardiac morphology and function. Therefore, further studies will be necessary to elucidate the underlying mechanisms associated to altered LV function in ART. Liu et al. (18) showed no signs of cardiac remodeling, however, significantly lower values in systolic and diastolic function in ART subjects were reported. This included significantly lower values for peak longitudinal strain but no alterations in EF (18). The assessment of longitudinal strain by 2DSTE is an emerging tool that can detect early and subtle changes in myocardial function even when EF is preserved (32). In our study, GPLS was comparable between both groups, bringing the reason for alterations in M-Mode assessed EF into question. Overall, M-Mode echocardiography in parasternal long axis view is assumed to reflect LV radial wall motion whereas longitudinal displacement is rather represented by 2DSTE in A4C (33). Radial function is dependent on the contraction of circumferential myocardial fibers while longitudinal function is facilitated by subendocardial fibers (34). In this study, no differences in longitudinal function visualized by GPLS were assessed in two, three or four chamber view. In addition, MAPSE, another parameter of LV longitudinal function, did not differ significantly between both groups. Therefore, the differences in M-Mode assessed EF and FS between ART and control subjects might be based on alterations in radial function. Still, the clinical relevance of these findings has to be further investigated as only differences within a normal reference range were displayed. Finally, Zhou et al. (17) described an increase in diastolic dysfunction and cardiac remodeling but did not report differences in LV systolic function. No differences were detected for EF when assessed in A4C (17). This supports that LV systolic function might not always be affected in ART subjects. For instance, in 50 % of patients with pressure overload, LV diastolic dysfunction evolves while systolic EF can be preserved (16). Furthermore, it is assumed that LV diastolic dysfunction often precedes unfavorable changes within LV systolic function (15, 16). Potentially – due to the relatively young cohort with an average age of 4 years – LV systolic alterations might not have become evident yet in the study conducted by Zhou et al. (17). The results in this presented study indicate a slight difference in M-Mode assessed EF and FS. However, after the adjustment for birth weight P. and gestational age, M-Mode assessed EF and FS displayed no significant differences between both groups. In addition, the remaining LV functional parameters were comparable between both groups. We assumed more profound changes in systolic function might manifest over time and conducted a correlation analysis between age and EF assessed through M-Mode and 2DSTE within both groups. Interestingly, no significant correlations were revealed within the ART cohort suggesting that LV systolic function does not worsen throughout childhood and adolescence. Nevertheless, given the relatively young cohort in this study, a longitudinal approach to evaluate cardiovascular morbidity, including LV systolic function, in ART subjects at advanced age is necessary.

#### Pathophysiologic considerations

The hypothesis of fetal programming of cardiovascular disease describes the influence of prenatal environmental conditions on the cardiovascular phenotype and the potential onset of chronic diseases later on in life (35). This begins with the early embryo which displays a particular vulnerability to environmental changes possibly leading to modifications in the embryonic development (10). Within the conceptional period, multiple modifications are implemented in the epigenome which increase the susceptibility for dysregulation (6, 36). Based on that, ART might represent a perturbance in the fetal environment leading to epigenetic and cardiovascular alterations. Fetal programming might also apply to children that suffered from fetal growth restriction and low birth weight as they display similar structural and functional changes within the cardiovascular system (e.g., cardiac remodeling, LV systolic and diastolic dysfunction) (37). Furthermore, prematurity might be associated with hypertrophied cardiomyocytes potentially resulting from premature exposure to pressure overload (38). ART is accompanied with a high prevalence of prematurity (10.9 % in singletons, ≥ 57.6 % in multiple pregnancies) and thus low birth weight (39). These factors might additionally impair the offspring’s cardiovascular function (5). In this study, LV systolic function displayed no significant differences between both groups when adjusted for birth weight P. and gestational age. This underlines the potential impairment of cardiac function due to perinatal risk factors. Preterm birth is considered to be a major risk factor for cardiovascular disease visualized by a higher prevalence of ischemic heart disease, hypertension or diabetes in affected patients (40). Significant differences in cardiac morphometry, diastolic function and systolic function, including EF and FS, can already be observed between preterm and full term neonates (41). This emphasizes the inverse relationship between preterm birth and cardiovascular function which might have had an impact on the outcome of the present study as well. Hence, further research is required to elucidate the individual and combined effect of prematurity and ART on overall cardiovascular health. Ultimately, genetic predispositions resulting from parental risk factors might contribute to an unfavorable phenotype in ART subjects (5). However, in animal studies vascular dysfunctions were still revealed even when ART mice without cardiovascular risk profile were examined (42). Certainly, further research is required to investigate which critical factors, if any, might lead to an increased cardiovascular morbidity, including an altered LV function, within the ART population.

## Limitations

### Study design

For the interpretation of the presented results, certain limitations should be taken into consideration: Participants for this study were matched by age and sex, nevertheless lifestyle factors and socioeconomic backgrounds might have influenced the outcome of this study. Further, perinatal conditions such as prematurity, presence of multiple pregnancy or maternal risk factors were purposely not defined as exclusion criteria for study participation. A prior exclusion of respective participants was considered to positively influence the “real” cardiovascular risk profile of the studied ART population. Information on pregnancy and birth was provided from clinical records and by questioning the participants’ parents. The retrospective data assessment on pregnancy and birth resulted in some loss of information. This study purposely included study participants ranging from children to young adults aiming to evaluate the influence of developmental stages on LV systolic function. However, as this study protocol did not apply a longitudinal approach, statements about the influence of age should be interpreted with caution. This study was limited by its sample size as ART study participants were recruited from one fertility center. In addition, ART procedures might differ between centers and new technologies are established over time. Therefore, multi-centric studies might be necessary for a more precise cardiovascular risk stratification and a better control of confounders. As cardiovascular disease might aggravate over the life span, future studies assessing clinical end points in adult study participants are needed for further risk stratification. The underlying mechanisms leading to cardiovascular alterations in ART subjects are diverse and might be based on a combination between epigenetic alterations, perinatal risk factors and vascular dysfunction. Therefore, molecular experiments might further elucidate the potential role of ART in cardiovascular health. Given the immense epidemiological impact of ART, future long-term multi-centric studies are required to assess cardiovascular morbidity, including LV systolic function, in ART subjects at advanced age.

### Assessment of left ventricular function

In this study, M-Mode echocardiography and semiautomatic LV volume and EF assessment through 2DSTE in A4C were conducted. Interestingly, a significant difference for EF was only displayed between both groups when M-Mode echocardiography was applied. In addition, EF tended to be higher when assessed through M-Mode echocardiography compared to semiautomatic assessment through 2DSTE in A4C. In the literature, an overestimation of M-Mode assessed EF is described when compared to an automated biplane or visual assessment modality (43). On the other hand, semiautomatic assessment through 2DSTE displayed low intra- and interobserver variability, nevertheless demonstrated an underestimation of EF when compared to real time three-dimensional echocardiography (RT3DE) (44). Despite M-Mode echocardiography being widely used, it shows a higher susceptibility of interobserver variability compared to an automated technology-based approach (45). In addition, M-Mode provides only limited information on LV contractility as only a single cross-section is analyzed (43). These factors should be taken into considerations as they might contribute to the discrepancies in EF results of the present study. In this study, relative intra- and interobserver variabilities of echocardiographic variables were considered to be adequate. Except for ESV (Teichholz) and SV (Teichholz), relative intra- and interobserver variabilities were < 10 %. In accordance with literature (44, 45), the provided data showed a tendency of higher relative observer variabilities for M-Mode echocardiography compared to 2DSTE. The use of RT3DE might have revealed more subtle alterations as it provides very accurate and reproducible measurements of LV volumes and EF (46). As cardiac magnetic resonance imaging is considered to be the gold-standard for the determination of LV volumetric and functional indices, it should be implemented in future study designs (47).

## Conclusion

This study suggests a significantly lower LV systolic function visualized by M-Mode echocardiography assessed EF and FS in subjects conceived through ART compared to spontaneously conceived peers. However, after the adjustment for birth weight P. and gestational age, M-Mode assessed EF and FS displayed no significant differences between both groups. The clinical relevance of these results has to be further evaluated as differences within a normal reference range were displayed, and LV systolic function parameters evaluated by other echocardiographic imaging modalities were comparable between both groups.

## Data availability statement

The original contributions presented in the study are included in the article/supplementary material, further inquiries can be directed to the corresponding author.

## Ethics statement

The studies involving human participants were reviewed and approved by Ethics Committee of the Medical Faculty of the Ludwig Maximilians University (LMU) Munich. All participants gave written informed consent before the examination. For minors, written informed consent was additionally received from parents or legal guardians.

## Author contributions

TV, AJ, NR, RDP, CT, NAH, and FSO: conceptualization. FS and FSO: methodology, software, validation, formal analysis, writing-original draft preparation, and visualization. AJ, NR, RDP, CT, NAH, and FSO: resources. FS, TV, MK, ML, BK, PL, AJ, NR, RDP, CT, NAH, and FSO: writing-review and editing. TV, AJ, NR, RDP, CT, NAH, and FSO: supervision. FS, TV, MK, ML, BK, PL, and FSO: project administration. All authors have read and agreed to the published version of the manuscript.

## Funding

This work was supported by the Deutsche Forschungsgemeinschaft (DFG, German Research Foundation) –413635475– and the Munich Clinician Scientist Program (MCSP) of the Ludwig Maximilians University (LMU) Munich.

## Conflict of interest

NR received support for symposium and others from Ferring Arzneimittel GmbH, Theramex Germany GmbH, Merck KGaA, Teva GmbH, Besins Healthcare.

The remaining authors declare that the research was conducted in the absence of any commercial or financial relationships that could be construed as a potential conflict of interest.

## Publisher’s note

All claims expressed in this article are solely those of the authors and do not necessarily represent those of their affiliated organizations, or those of the publisher, the editors and the reviewers. Any product that may be evaluated in this article, or claim that may be made by its manufacturer, is not guaranteed or endorsed by the publisher.
